# Combined effects of inflorescence developmental age and environmental conditions during microgametogenesis on pollen viability, germination and size in *Musa acuminata* ssp.

**DOI:** 10.1093/aob/mcaf268

**Published:** 2025-10-23

**Authors:** Astrid Severyns, Rony Swennen, Steven B Janssens, Nico De Storme

**Affiliations:** Laboratory of Plant Genetics and Crop Improvement, Division of Crop Biotechnics, Department of Biosystems, KU Leuven, Heverlee 3001, Belgium; Department of Research, Meise Botanic Garden, Meise 1860, Belgium; Leuven Plant Institute, KU Leuven, Heverlee 3001, Belgium; Laboratory of Tropical Crop Improvement, Division of Crop Biotechnics, Department of Biosystems, KU Leuven, Heverlee 3001, Belgium; International Institute of Tropical Agriculture, Banana Breeding, Namulonge-Sendusu, Box 7878, Kampala 25601, Uganda; Department of Research, Meise Botanic Garden, Meise 1860, Belgium; Leuven Plant Institute, KU Leuven, Heverlee 3001, Belgium; Department of Biology, KU Leuven, Heverlee 3001, Belgium; Laboratory of Plant Genetics and Crop Improvement, Division of Crop Biotechnics, Department of Biosystems, KU Leuven, Heverlee 3001, Belgium; Leuven Plant Institute, KU Leuven, Heverlee 3001, Belgium

**Keywords:** Pollen viability, pollen germination, pollen size, inflorescence, developmental age, microgametogenesis, environmental conditions, *Musa acuminata* ssp

## Abstract

**Background and Aims:**

Male fertility is often suboptimal in parthenocarpic banana cultivars, hindering their use as male parents in current banana breeding schemes. Next to genotype-specific traits, such as ploidy and structural heterozygosity, inflorescence developmental age and environmental factors can significantly influence male reproductive performance.

**Methods:**

In this study, pollen viability, germination capacity and diameter, were closely monitored throughout the entire male flowering phase of *Musa acuminata* wild diploids and diploid and triploid cultivars. Additionally, environmental parameters, including temperature, light radiation and humidity, were monitored up to 40 days before anthesis to determine their influence on microgametogenesis.

**Key Results:**

Wild accessions showed a gradual reduction in pollen germination over the flowering period. High light radiation prior to pollen mitosis I and II reduced germination capacity, whereas during meiosis it negatively affected pollen viability. Conversely, in cultivated bananas, pollen traits improved with the developmental age of the male inflorescence. In these cultivars, higher temperatures during meiosis enhanced pollen diameter, viability and germination rates.

**Conclusions:**

These findings indicate that both inflorescence maturity and specific environmental conditions during pollen development significantly influence pollen quality in bananas. Pollen performance could be optimized in wild diploids by obtaining pollen in the first months of the male flowering phase and shielding the plants from high light intensities, whereas for cultivars the pollen should be harvested towards the end of the male flowering phase, and a heat treatment during meiosis could lead to higher pollen quality and thereby increase breeding success.

## INTRODUCTION

Bananas and plantains are among the most important crops in the world, together good for a production of almost 180 million tons in 2022, and being consumed by >400 million people daily (Brown *et al.*, 2017; Crop Data: Banana, 2022). Any other crop of this importance has shifted towards hybrid breeding in the last few decades; however, for banana barely any improved varieties are currently in use. Banana breeding programmes have been active over the last 100 years but are hindered by low seed yield and viability (Vuylsteke *et al.*, 1993; Batte *et al.*, 2019). Despite the relevance of reproductive barriers in banana breeding, many factors in the pollination and subsequent process of seed formation remain insufficiently characterized. Suboptimal pollen and ovule viability related to genomic imbalances in parental lines, low receptivity of the female stigma, poor timing of pollination, lack of pollen tube growth and fertilization of ovules, and post-fertilization growth arrest in embryos all contribute to the failure of seed set in banana cultivars. However, the nature and relative share of these defects in the ultimate seed formation bottleneck remain to be determined (Uma and Arun, 2016; Amah *et al.*, 2021).

Potential fertilization in flowering plants is *a priori* related to the capacity of pollen to germinate, thereby providing male sperm cells via the elongation of pollen tubes towards the embryo sac (Taylor and Hepler, 1997). Pollen germination is strongly influenced by external factors such as temperature, humidity and time of day, and by physiological factors, such as successful anther dehiscence, pollen quantity and quality, stigma receptivity, compatibility and enzymatic activity of stigmas and growing pollen tubes (Byers, 1995; Cheung, 1995; Sanzol *et al.*, 2003; Boavida and McCormick, 2007; Distefano *et al.*, 2012). Also, during early development of male gametophytes, factors such as temperature, drought stress and plant nutrition play an important role, because these conditions can lead to male sterility (Ito *et al.*, 1970; Saini *et al.*, 1984; Endo *et al.*, 2009). Accordingly, previous research on pollen viability and germination capacity in banana has focused on separate interfering factors as conferred by differences in genotype, ploidy level, plant developmental age or environmental influences (Ortiz *et al.*, 1998; Adeleke *et al.*, 2004; Fortescue and Turner, 2004; Ssebuliba *et al.*, 2008; Goigoux *et al.*, 2013). An interpretation of the combined effect of these factors has not yet been performed.

Numerous studies in *Musa* have assessed pollen viability and fertility across genotypes, revealing noteworthy variations at inter- and intraspecific levels and at different ploidy levels within the *Musa acuminata* background (Adeleke *et al.*, 2004; Fortescue and Turner, 2004; Soares *et al.*, 2008; Mukasa and Rubaihayo, 1993; Bayo *et al.*, 2024). According to Bayo *et al.* (2024) and Fortescue and Turner (2004), wild *M. acuminata* accessions (AA) show high pollen viability rates of ∼80 %, using tetrazolium chloride stain and Alexander’s stain, respectively, whereas both studies found reduced fertility in diploid cultivars (AAcv), with 40–60 % viable pollen. This variance is often attributed to the genomic make-up of parental lines and, in particular, to the interaction between contributions from multiple *M. acuminata* subspecies, resulting in structural heterozygosity (Shepherd, 1999; Goigoux *et al.*, 2013; Šimoníková *et al.*, 2020; Beránková *et al.*, 2024). Triploid plantains (AAB) and East African Highland bananas (AAA), in contrast, typically show a high gametophytic sterility, with only 3–13 % pollen viability (Fortescue and Turner, 2004), which is attributed to their uneven number of chromosomes leading to irregularities in meiotic cell division and genomic imbalances in microgametogenesis (Shepherd, 1999).

Banana is protogynous, and its large conical inflorescence consists of enveloping bracts that contain clusters of ∼20 flowers. The inflorescence meristem continuously produces new flower primordia and is located in the centre of the cone. Towards the exterior of the cone, increasingly mature flowers are found, up to fully mature flowers in anthesis on the outside of the bud. Bracts open consecutively at an average rate of one to two per day, starting with 4–12 female flower clusters depending on the genotype, after which the meristem differentiates to male flower clusters, often with the formation of a few infertile or hermaphrodite flower clusters in between (Turner *et al.*, 2024). Female flowers clusters remain attached to the stem to develop further into fruits in parthenocarpic genotypes and in wild genotypes if pollinated, whereas male flower clusters abscise 1–2 days after anthesis, leaving behind a scar. In general, the inflorescence continues to produce male flowers until fruit maturity and stem withering (Bakry *et al.*, 2009). Overall, banana is outcrossing, except for some accessions with functional hermaphrodite flowers (Kallow *et al.*, 2021). Outcrossing plants are known to show some degree of reproductive senescence (Malagon *et al.*, 2019), and with *Musa* plants generating male flowers for several months, pollen performance might deteriorate throughout the flowering period. Krishnakumar *et al.* (1992) were first to note that *in vitro* pollen germination in *Musa* AA diploid cultivars is influenced by the age of the male bud, with weak germination of pollen obtained from the first male flowers, maximal pollen performance of flowers between clusters 20 and 30, and a gradual decline of germination capacity in the subsequent clusters. Likewise, a study by Ssebuliba *et al.* (2008) on triploid East African Highland bananas and the diploid cultivar ‘Pisang Lilin’ showed that flowers between clusters 30 and 40 exhibited the highest pollen viability according to acetocarmine stain. In accordance, Goigoux *et al.* (2013) reported a decline of pollen viability during male inflorescence development in ‘Mchare’ diploid cultivars via Alexander’s stain. In contrast, male bud age did not affect pollen viability in the wild diploid accession ‘Calcutta 4’, which showed high and uniform pollen viability along its complete rachis (Ssebuliba *et al.*, 2008). Pollen viability in banana, as in many other plant species, is also significantly influenced by several environmental parameters. For a set of AA diploids, Ortiz *et al.* (1998) found that high solar radiation, high temperature, low relative humidity (RH) and low rainfall enhanced pollen viability (acetocarmine stain), whereas high rainfall and high minimum relative humidity reduced it. Uma and Arun (2016) confirmed that higher temperatures resulted in increased *in vitro* germination rates, although temperatures of >35 °C tended to decrease the germination percentage.

Next to pollen viability and germination, pollen size is another parameter relevant to overall pollen quality, which is also influenced by both intrinsic and external factors throughout microgametogenesis. Pollen smaller than the standard haploid pollen grain size indicates defects in pollen grain formation, resulting in small, infertile, aborted pollen (Ascari *et al.*, 2020; Du *et al.*, 2023). On the contrary, pollen larger than the haploid size might indicate the formation of pollen with a higher ploidy level, such as diploid (2*n*) pollen through meiotic restitution, a common phenomenon in plants (Brownfield and Köhler, 2011). When the number of chromosomes in a cell increases, its nucleus and cytoplasm will expand (Darlington, 1937; Karcz *et al.*, 2000). Previous research has linked a rise in pollen diameter to an increase in pollen ploidy in bananas (Sathiamoorthy, 1994; Ortiz, 1997; Tenkouano *et al.*, 1998). Findings by Ortiz *et al.* (1998) revealed that the production of 2*n* pollen in banana is positively associated with solar radiation, pan evaporation and temperature and negatively associated with rainfall and minimum RH.

Although various *ad hoc* studies have examined pollen viability, germination capacity and pollen size in banana, particularly in comparisons between different genotypes, no comprehensive study has yet explored how these parameters are impacted by the developmental age of the male flower as combined with environmental shifts occurring during male gametogenesis. In this study, we addressed this by monitoring the effect of the aforementioned factors and their putative interaction on pollen viability, germination capacity and size in a diverse set of banana genotypes, including wild diploids, a cultivated diploid and cultivated triploids.

## MATERIALS AND METHODS

### Biological material

Two types of plant material were collected, i.e. flowers in anthesis containing mature pollen that were used fresh for pollen viability, germination and size assessments immediately after harvest, and male flowers in different stages of microgametogenesis that were fixed for later nuclear staining.

#### Fresh mature flowers for pollen viability, germination and size assessments

**Fig. 1.**
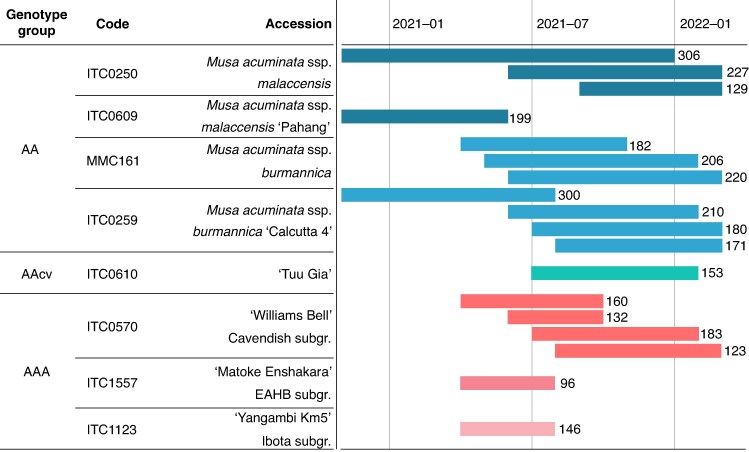
Different *Musa* accessions used in this study, including their genotypic group (AA, AAcv or AAA), reference code and full accession name. Each bar denotes a separate male inflorescence and the time interval during which it was monitored. The number at the right of each bar represents the total number of male nodes formed throughout the flowering period of that inflorescence.

From November 2020 to February 2022, fresh male flowers were sampled throughout male flowering from several banana inflorescences; i.e. per inflorescence, the new flower cluster in anthesis was harvested between 08:30 and 09:30 h every 2 weeks from the start until the end of the male flowering phase. During each sampling, the age of the inflorescence was determined by counting the number of male scars on the inflorescence rachis. This was done for two different accessions of both *Musa acuminata* ssp. *malaccensis* and *Musa acuminata* ssp. *burmannica* wild diploids (AA), ‘Tuu Gia’ as a diploid cultivar (AAcv), and triploid cultivar (AAA) accessions from three genetically different subgroups, namely ‘Williams Bell’, ‘Matoke Enshakara’ and ‘Yangambi Km5’ (Fig. 1). All plants were grown in full soil inside the same compartment of a greenhouse facility located in Heverlee, Belgium (50°N). Afterwards, the frequency of flower opening could be calculated by dividing the total number of days of the flowering period by the total number of scars on the rachis.

#### Fixation of male flowers for assessment of microgametogenesis

To determine the time frame of microspore development in *M. acuminata* ssp., one *M. acuminata* ssp. *malaccensis* and one *M. acuminata* ssp. *burmannica* ‘Calcutta 4’ male flower bud were harvested in the KU Leuven greenhouse on 16 November 2020. The male flower buds were dissected by separating all enveloping flower clusters that it contained. Following the developmental gradient from the mature flower clusters at the exterior of the bud towards the flower clusters undergoing meiosis at the interior of the bud, the entire microgametogenesis of banana can be found. Every five flower clusters from the cluster in anthesis at the exterior of the bud, an entire cluster was fixed in Clarke’s fixative (75 % ethanol and 25 % acetic acid) to allow later cytological examinations. Each flower cluster contained ∼20 flowers. Following overnight fixation, the flower clusters were stored in 70 % ethanol at 4 °C. To allow additional observations of mature pollen nuclei at a different location and different time point, flower clusters in anthesis of *M. acuminata* ssp. *malaccensis*, *M. acuminata* ssp. *burmannica* ‘Calcutta 4’ and diploid AAcv cultivars ‘Pitu’, ‘Malaysian blood’ and ‘Uwati’ were harvested from plants in the field in Arusha, Tanzania (3°S), on 27 September 2021. The average temperature in Arusha in September 2021 varied around 20 °C, and the RH was ∼70 %. Solar radiation was not recorded. Following dissection of the anthers out of the flowers, they were fixed as described above.

### Greenhouse conditions

Environmental conditions in the greenhouse were controlled by an automated system (Table 1). Additional lighting was provided by high-pressure sodium lamps (SON-T, Philips). Plants received nutrients (Supplementary Data Table S1) and water multiple times per day via an automated dripping system.

**Table 1.** Greenhouse compartment settings.

Variable	Timing	Set value
Heating	06:00–22:00 h	26 °C
	22:00–06:00 h	16 °C
		Gradient, 1 °C per 15 min
Ventilation	When temperature = set + 0.5 °C	Open windows
Lighting	06:00–22:00 h when natural light <450 W m^−2^	25 W m^−2^ additional
Relative humidity	Continuous	80 %
	Windows open	<80 %

### Environmental measurements

**Fig. 2.**
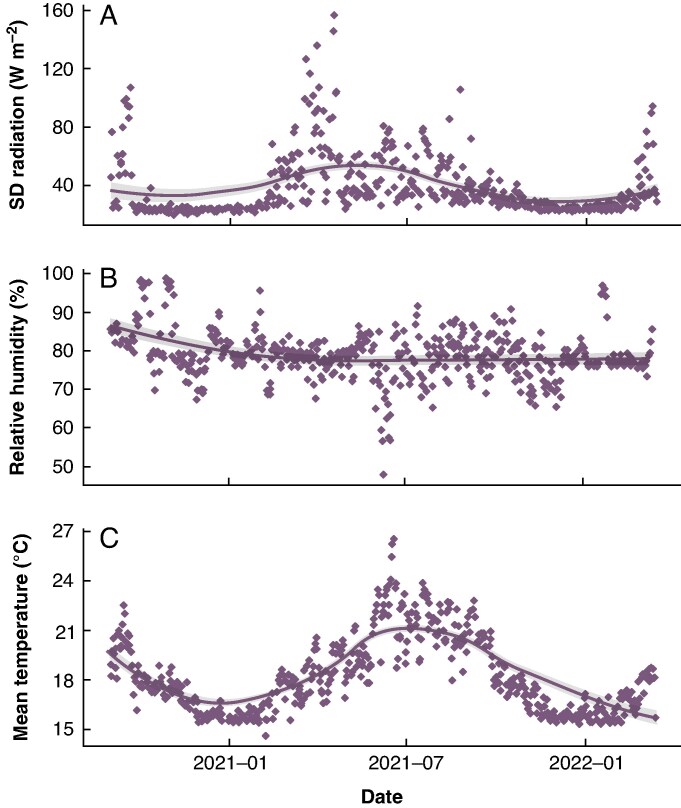
Daily measurements of: (A) s.d. of light radiation intensity (in watts per metre squared); (B) mean relative humidity (as a percentage); and (C) mean ambient temperature (in degrees Celsius) in the greenhouse from November 2020 to February 2022. A smooth loess curve, with 95 % confidence interval in grey, was fitted to the general trend in the data.

Every 5 min, light radiation (solar and artificial) was measured by a Quantum sensor (Sky instruments), and RH and temperature levels were recorded by a Box psychrometer (Priva). The daily s.d. in radiation intensity ranged from 20 to 160 W m^−2^, with a mean of 38 ± 21 W m^−2^ (Fig. 2A). Mean daily RH fluctuated around 80.0 ± 6.5 % but occasionally dropped to 50 % during summer months (Fig. 2B). Mean daily temperature was 18.2 ± 2.2 °C, although peaks of 35 °C were reached in summer and minimum values of 16 °C during winter (Fig. 2C).

The greenhouse climate in this study mimicked a humid subtropical climate that is found between 20° and 35° N and S latitude, with an average temperature of 27 °C during the warmest months and on average 12 °C during the coldest months, in which bananas can be managed successfully (Saúco Galán *et al.*, 2012). In comparison to an annual average solar irradiance of 200–220 W m^−2^ in the subtropics (Loster, 2010), plants in the greenhouse received on average 220 W m^−2^ during summer; during winter, however, even with additional lighting, they received only 50 W m^−2^ on average. The latter is a major difference from the natural growth conditions of banana and should be taken into account when interpreting the results of this study.

### Assessment on fresh mature pollen of pollen viability, germination and size

Every 2 weeks during flowering of the genotypes listed in Table 1, fresh male flower clusters in anthesis were harvested, after which pollen germination, viability and size were assessed immediately through *in vitro* pollen germination, fluorescein diacetate (FDA) staining and bright-field microscopy.

#### Pollen germination

The medium for *in vitro* assessment of pollen germination was prepared according to Brewbaker and Kwack (1963), with 0.01 % boric acid, 0.01 % potassium nitrate, 0.02 % magnesium sulphate, 0.03 % calcium nitrate, 10 % sucrose and 0.5 % plant agar, brought to a pH of 6.0. After autoclaving, 100 µL of the medium was spread over a microscopy slide and left to solidify for 5 min. Pollen of a freshly harvested flower was then picked up with a brush and distributed evenly over the medium patch. For each inflorescence, this was done for three different flowers per cluster. Next, the slides were placed inside a humid chamber and kept for 24 h at 25 °C in darkness (Nyine and Pillay, 2007; Soares *et al.*, 2008). After overnight incubation, pollen germination was assessed quantitatively via bright-field microscopy (Olympus, Bx40) by counting the total number of pollen grains and the number of pollen tubes in four random 40× microscopy images per slide. Following Soares *et al.* (2008), protrusions shorter than the pollen grain diameter were not counted, under the assumption that truly elongating pollen tubes would have progressed beyond this distance after 24 h.

Pollen germination rate was calculated as:


$$\mathrm{Pollen}\;\mathrm{germination}\;\mathrm{rate}\;(\text{\%})=\frac{\mathrm{Number}\;\mathrm{of}\;\mathrm{pollen}\;\mathrm{tubes}}{\mathrm{Total}\;\mathrm{number}\;\mathrm{of}\;\mathrm{pollen}\;\mathrm{grains}}\times 100$$

#### Pollen size and viability measurements

Pollen grains were stained with FDA to assess their metabolic activity as a measure for pollen viability. For each inflorescence, pollen from three anthers from three different flowers within a cluster were taken with forceps, transferred to 1 mL of FDA staining solution and incubated for 5 min. The staining solution consisted of 900 µL of 10 % sucrose and 100 µL of 2 mg FDA per 1 mL acetone solution, with a final concentration of 0.2 mg FDA mL^−1^ (Heslop-Harrison and Heslop-Harrison, 1970). After centrifugation of the pollen solution at 30 *g* for 2 min, the supernatant was discarded. The pollen pellet was resuspended in 100 µL of 10 % sucrose solution and pipetted onto a microscopy slide. The pollen was imaged with a Leica DMi 8 microscope in bright-field mode to assess pollen size and in the Green Fluorescent Protein channel (450–490 nm) to measure FDA intensity. LAS X software was used to capture 18 images per microscopy slide automatically.

To identify pollen grains and perform size and FDA intensity measurements in a high-throughput manner, an image analysis pipeline was customized in CellProfiler software (version 4.2.4) (Lamprecht *et al.*, 2007). This pipeline outputs .csv files with various size and fluorescent intensity parameters, of which the equivalent diameter and mean intensity were used in further analyses. To obtain robust pollen viability percentages based on the FDA staining assay, resulting .csv files were analysed using Rstudio software (R Studio Team, 2021). Pollen FDA intensity histograms typically showed two peaks: one at low fluorescence, representing dead pollen; and one at higher FDA intensity, indicating viable pollen. For each pollen sample, a kernel density function was used to fit a distribution on the FDA intensity histograms. Next, the R package ‘*multimode*’ was used to identify the exact position of the antinode between low and high FDA intensity; this value was used as a threshold to distinguish dead from viable pollen (Supplementary Data Fig. S1).

Pollen viability was then calculated as follows:


$$\begin{array}{r} \mathrm{Pollen}\;\mathrm{viability}\;(\text{\%})=\frac{\mathrm{Amount}\;\mathrm{of}\;\mathrm{pollen}\;\mathrm{with}\;\mathrm{FDA}\;\mathrm{intensity}\;\mathrm{value}\;\mathrm{exceeding}\;\mathrm{the}\;\mathrm{set}\;\mathrm{threshold}}{\mathrm{Total}\;\mathrm{amount}\;\mathrm{of}\;\mathrm{identified}\;\mathrm{pollen}}\times 100 \end{array}$$

### DAPI staining of fixed microspore and pollen nuclei

To determine the developmental state of microspores obtained from flower clusters at different stages throughout male bud development, their nuclear configuration was monitored using DAPI staining. Three anther pieces of length 1 mm were dissected out of three different flowers of each fixed cluster and were placed in 100 µL of DAPI staining solution, consisting of 1 mg DAPI, 1 mL EDTA and 20 µL Triton X-100 per 20 mL of 1× phosphate buffer (pH 7.4) (Park *et al.*, 1998). Anther pieces were incubated in this staining solution for 1 h at 60 °C after which they were placed on a microscopy slide and squashed with a dentist’s hook. Finally, slides were imaged with a DMi8 microscope (Leica) using the LED405 channel (375–435 nm).

### Statistical analysis

To compare the different pollen quality parameters, percentage viability, percentage germination and mean pollen diameter between genotypes, Kruskal–Wallis χ^2^ tests and *post hoc* Wilcoxon tests were performed in Rstudio (R Studio Team, 2021).

The effect of inflorescence age on the different pollen quality parameters was assessed with a linear mixed model using the R package *lme4* (Bates *et al.*, 2015; Pinheiro *et al.*, 2023). The relative developmental age of each flower cluster was determined by dividing the number of scars that were present on the rachis when the cluster was harvested by the total number of scars at the end of male flowering, resulting in a scale ranging from 0 to 100. The model included genotype as a categorical random effect predictor and the relative developmental age (age) and its quadratic term (age^2^) as continuous fixed-effect predictors. Backward stepwise regression was performed to analyse whether age and age^2^ had significant effects. If not, the model was re-run without insignificant term(s), and coefficient estimates were obtained for the remaining parameters.

Environmental parameters up to 40 days prior to the pollen evaluation measurements were also added to the models as continuous fixed-effect predictors. Care was taken to avoid multi-collinearity between parameters, because they can inherently relate to each other. Prior to running the models, parameters showing least correlation were chosen, namely, daily standard deviation in radiation (SD rad; in watts per metre squared), daily mean temperature (mean *T*; in degrees Celsius) and daily mean RH (as a percentage), with correlations of 0.53 between mean *T* and SD rad, −0.22 between mean RH and SD rad, and −0.17 between mean *T* and mean RH. Additionally, during the model-selection step, parameters with variance inflation factor above three were removed. Again, linear mixed models were constructed via a combination of backward and forward selection using the *lme4* package. Model quality was assessed by checking normality and homoscedasticity of the residuals and by conditional and marginal *R*^2^. Conditional *R*^2^ describes the proportion of variance explained by both fixed and random effects, whereas marginal *R*^2^ describes only the proportion of variance explained by fixed effects.

## RESULTS

### Genotypic differences in pollen viability, germination and pollen size

**Fig. 3.**
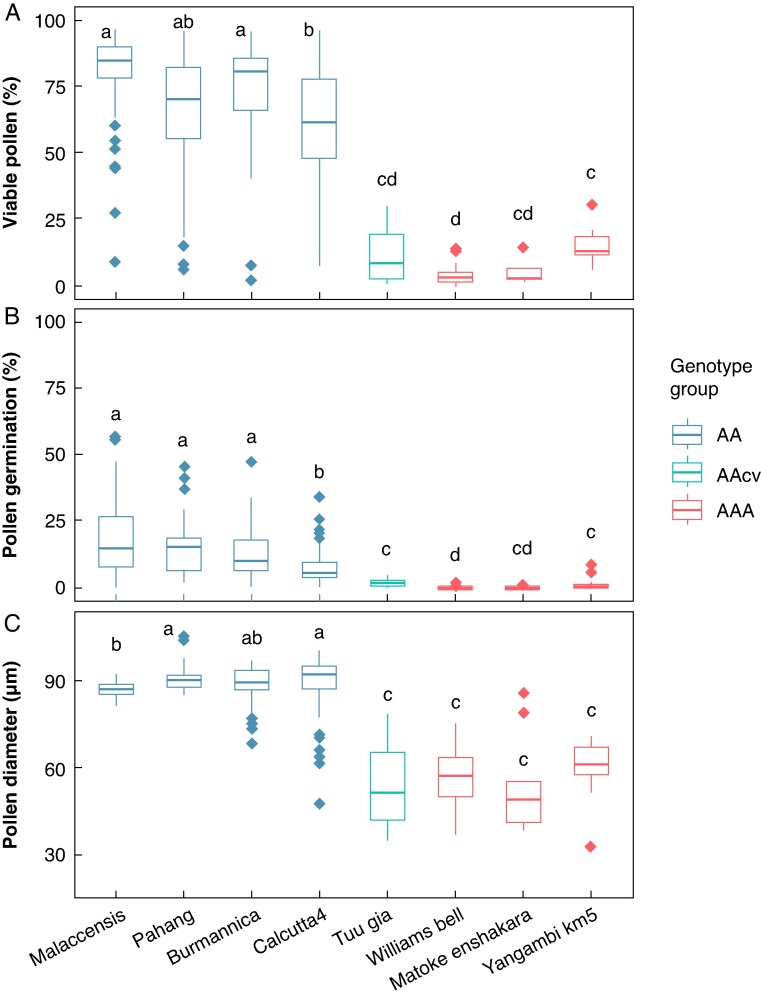
Pollen quality parameters for the assessed varieties. Boxplots of percentage viable pollen according to FDA staining intensity (A), percentage germination (B) and pollen diameter of mature pollen (C). Genotypes showing significant differences in each of these parameters according to *post hoc* Wilcoxon tests are indicated by different letters.

Large statistically significant differences were found between the percentages of viable pollen (χ^2^_7_ = 175.58, *P* < 2.2 × 10^−16^), percentages of pollen germination (χ^2^_7_ = 188.78, *P* < 2.2 × 10^−16^) and pollen diameters (χ^2^_7_ = 198.16, *P* < 2.2 × 10^−16^) of the genotypes included in this study. There was a clear separation between wild diploids and the diploid and triploid cultivars, with wild diploids showing significantly higher pollen viability, germination and pollen diameters than cultivars (Fig. 3). Therefore, the data were split into ‘wild’ and ‘cultivar’ for subsequent model construction to have two more homogeneous datasets that allow more accurate prediction within each of the groups.

### Dynamics of pollen performance parameters over inflorescence development

**Fig. 4.**
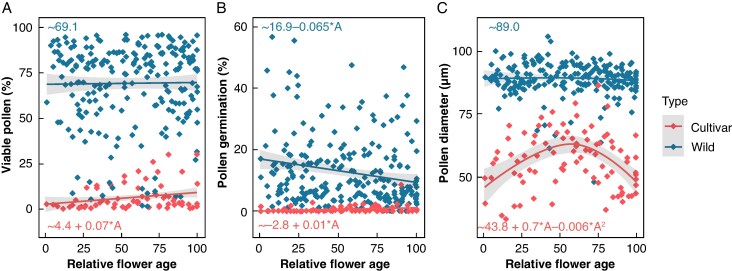
Temporal dynamics of pollen viability (A), germination rate (B) and pollen diameter (C) throughout male inflorescence development in *Musa* wild varieties and cultivars. The curves represent the linear mixed models fitted through the data points, with the grey area surrounding the curves indicating the 95 % confidence interval of predictions. Model formulas, with ‘A’ denoting the relative developmental age, and ‘A^2^’ its quadratic term, are shown in the graphs.

For both wild accessions and cultivars, variation in the percentage of viable pollen, the percentage of pollen germination, and pollen diameter over the relative developmental age of the inflorescence was evaluated through the construction of linear mixed models. Inflorescence age did not significantly affect mean pollen viability in wild accessions, with 69.1 ± 22.6 % viable pollen throughout flowering (Fig. 4A). In cultivars, however, the developmental age significantly impacted pollen viability, with a small positive coefficient of 0.07 (s.e. = 0.025, *P* = 0.00734), equal to an increase of 0.07 % of viable pollen per unit of age (Fig. 4A). Accordingly, the mean percentage of viable pollen corresponded to 4.4 % at age 0 (intercept) and increased to 11.2 % by the end of male flowering. Pollen germination was impacted significantly by the developmental age of the inflorescence in both wild accessions and cultivars. In cultivars, pollen germination was overall close to zero but with a small positive effect of age (*β* = 0.01, s.e. = 0.004, *P* = 0.0296), whereas in wild accessions germination was generally much higher, starting at 16.9 %, but was affected negatively by age (*β* = −0.065, s.e. = 0.022, *P* = 0.003978), gradually decreasing towards a mean germination of 11.6 % at the end of male flowering (Fig. 4B). The mean pollen diameter of wild accessions was highly stable throughout inflorescence development, with a consistent value of 89.0 ± 6.6 µm (Fig. 4C), indicating a uniform haploid pollen population. For cultivars, the mean diameter of pollen grains was much lower and showed high variability over male flowering, as a consequence of the many small and aborted pollen grains that were formed, next to a variable fraction of pollen with a regular ‘haploid’ (90 µm) or even ‘diploid’ diameter (120 µm) (Supplementary Data Fig. S2). In general, the mean pollen diameter in cultivars was found to follow a parabolic curve over the male flowering phase, with a positive coefficient of 0.671 (s.e. = 0.151, *P* < 0.0001) for age and with a negative coefficient of −0.006 (s.e. = 0.001, *P* < 0.0001) for age^2^. The optimum of well-formed and potentially viable pollen grains in cultivars thus occurs mid-male flowering, more specifically around inflorescence developmental age 60 (Fig. 4C).

### Time frame of male gametogenesis in *Musa*

Before adding the effect of environmental conditions during microgametogenesis to the models, we investigated where critical time points during male gametogenesis in *Musa* are situated relative to the moment of flower anthesis.

**Fig. 5.**
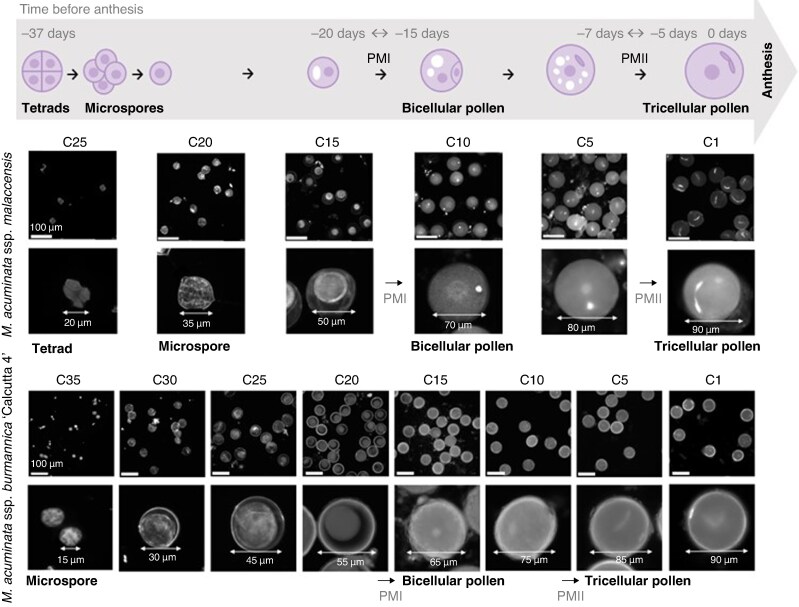
Time line of the occurrence of distinct microspore stages in *Musa accuminata* ssp. *malaccensis* and *M. accuminata* ssp. *burmannica* ‘Calcutta 4’. Images show representative nuclear configurations of DAPI-stained microspores isolated from flower clusters in different developmental stages, where C1 represents one flower cluster before anthesis, C5 represents five flower clusters before anthesis etc., until the respective end point of meiosis marked by tetrads (C25 *M. a.* ssp. *malaccensis*) or early microspores (C35 ‘Calcutta 4’). Abbreviations: PMI, pollen mitosis I; PMII, pollen mitosis II.

During the measuring period, *M. a.* ssp. *malaccensis* plants opened on average one flower cluster every 1.5 days, whereas for ‘Calcutta 4’, flower clusters opened daily (Supplementary Data Table S2). This indicates that ‘Calcutta 4’ inflorescences bear more flower clusters that are closer to each other in development in comparison to those in *M. a.* ssp. *malaccensis*. To confirm this, a *M. a.* ssp. *malaccensis* and ‘Calcutta 4’ male flower buds were dissected to isolate all developing flower clusters, starting from mature flowers at cluster one before anthesis to the end point of meiosis at clusters 25 and 35, respectively (Fig. 5). By correcting for one flower cluster opening every 1.5 days for *M. a.* ssp. *malaccensis*, the tetrad stage was calculated to take place 37 days prior to anthesis, closely coinciding with the early microspores found in ‘Calcutta 4’ at cluster 35 and thus 35 days prior to anthesis. With meiosis in banana during ∼2–3 days in total (Dodds and Simmonds, 1946), development from the pollen mother cell to mature pollen grain commences ∼40 days prior to anthesis. Nuclear staining of the developing microspores in the different flower clusters revealed that pollen mitosis I (PMI) occurs between flower clusters 10 and 15 in *M. a.* ssp. *malaccensis* and between clusters 15 and 20 in ‘Calcutta 4’, corresponding to a time frame between 15 and 20 days prior to anthesis (using the same correction factor for *M. a.* ssp. *malaccensis*). Pollen mitosis II (PMII) takes place between clusters 1 and 5 in *M. a.* ssp. *malaccensis* and between clusters 5 and 10 in ‘Calcutta 4’, implying that PMII takes place 5–7 days prior to anthesis.

To verify that PMII in *M. a.* ssp. *malaccensis* and ‘Calcutta 4’ consistently happens prior to flower anthesis, mature pollen from plants grown under different climatic circumstances in the field in Arusha, Tanzania, were subjected to nuclear staining, and although the more faint vegetative nucleus was not always visible, the germ unit was clearly composed of two separate vegetative nuclei (Supplementary Data Fig. S3A–H). Similar tricellular arrangements were visible in mature pollen of AAcv cultivars ‘Pitu’, ‘Malaysian blood’ and ‘Uwati’ (Supplementary Data Fig. S3I–T).

Overall, these data show that male gametogenesis starts as early as 40 days prior to anthesis. This is why, under the assumption of uniformity in the progression of microgametogenesis across wild *M. acuminata* ssp. and their derived AAcv and AAA cultivars, in the following analyses the environmental conditions 40 days prior to anthesis were included in the modelling of the three pollen quality parameters.

### Impact of environmental conditions during gametogenesis on pollen viability, germination rate and pollen size

Taking advantage of the extensive data gathered on pollen size and pollen performance parameters from different *Musa* inflorescences that developed over a range of environmental conditions, putative effects of environmental conditions occurring during male gametogenesis (i.e. until 40 days before anthesis) on pollen viability, germination and size were determined via models. In this modelling, a distinction was made between wild *M. acuminata* accessions and cultivars.

The model predicting the percentage of viable pollen for wild *M. acuminata* accessions (Table 2) displayed a mediocre fit, with random and fixed effects combined accounting for 0.268 of variation (conditional *R*^2^) and with fixed effects accounting for 0.105 of variation (marginal *R*^2^). Although environmental conditions thus play a modest role here, radiation and temperature had significant impacts. More specifically, the radiation level during meiosis (−37 days) and 3 days before anthesis had a negative effect on the percentage of viable pollen. On the contrary, the light radiation level immediately before the first pollen mitotic division (−21 days) was found to have a positive effect on pollen viability. A higher temperature 10 days prior to anthesis also had a considerable positive effect on viability.

**Table 2.** Coefficient estimates for linear mixed models explaining the variability in pollen viability and pollen germination rate for wild *Musa* accessions.

Response	Term	Estimate	s.e.	d.f.	*t* value	Pr(>|*t*|)
Percentage viable pollen	Intercept	40.056	10.522	65.645	3.807	0.000312
	s.d. rad −3 days	−0.326	0.069	221.601	−4.714	4.29 × 10^−6^
	Mean *T* −10 days	2.045	0.602	221.902	3.397	0.000807
	s.d. rad −21 days	0.320	0.089	221.178	3.570	0.000438
	s.d. rad −37 days	−0.193	0.091	221.237	−2.116	0.035462
Pollen germination percentage	Intercept	24.919	11.771	215.540	2.117	0.035417
	Age	−0.082	0.020	227.756	−4.070	6.50 × 10^−5^
	Mean *T* −6 days	2.788	0.362	227.557	7.706	4.00 × 10^−13^
	s.d. rad −10 days	−0.342	0.048	227.380	−7.170	1.04 × 10^−11^
	Mean RH −14 days	−0.287	0.076	227.183	−3.755	0.000221
	s.d. rad −24 days	−0.169	0.049	227.150	−3.473	0.000616
	Mean RH −37 days	−0.214	0.095	227.297	−2.262	0.024620

Abbreviations: Age, relative inflorescence developmental age; s.d. rad, mean standard deviation in light radiation; mean *T*, mean temperature; mean RH, mean relative humidity, followed by the day respective to the moment of flower sampling on which the conditions took place.

The model for pollen germination in wild accessions (Table 2) showed a better fit to the data, with random and fixed effects combined explaining nearly half of the observed variation (conditional *R*^2^ of 0.413). Fixed effects alone accounted for a marginal *R*^2^ of 0.243. Also here, several environmental factors occurring during microgametogenesis were found to affect pollen germination rate; in particular, radiation and mean RH had significant impacts. Germination was negatively influenced by high light radiation occurring 24 and 10 days before anthesis, corresponding to the developmental stages immediately preceding PMI and PMII, respectively. Additionally, RH at 37 days (during meiosis) and 14 days before anthesis (i.e. between PMI and PMII) negatively impacted the germination capacity of future pollen grains. Finally, temperature also influenced pollen germination rate, with especially higher temperatures occurring during PMII (−6 days) having a large positive effect.

Given that pollen diameters were highly uniform in wild accessions, no parameters were discovered that could explain variability in this trait, hence no model was constructed.

For the *Musa* cultivar group, the model explaining variation in pollen viability showed a relatively good fit when taking into account both random and fixed variables, with conditional *R*^2^ of 0.611. This, however, dropped to a marginal *R*^2^ of 0.184 when considering only fixed effects, indicating that a substantial part of the observed variation was attributed to the random effect of cultivar genotype. Still, a few environmental effects were uncovered, with pollen viability showing a positive relationship to the ambient temperature and RH occurring at 37 days before anthesis, corresponding to final phases of meiosis (Table 3).

**Table 3.** Coefficient estimates for linear mixed models explaining the variability in pollen viability, pollen germination rate, mean pollen diameter and associated diameter standard deviation in the tested *Musa* cultivars.

Response	Term	Estimate	s.e.	d.f.	*t* value	Pr(>|*t*|)
Percentage viable pollen	Intercept	−47.271	11.829	64.904	−3.996	0.000167
	Age	0.102	0.023	62.297	4.490	3.14 × 10^−5^
	Mean T −37 days	1.084	0.314	62.467	3.451	0.001006
	Mean RH −37 days	0.366	0.120	62.864	3.038	0.003468
Pollen germination percentage	Intercept	−1.919	0.968	56.537	−1.983	0.05228
	Age	0.010	0.004	83.628	2.448	0.01646
	Mean *T* −40 days	0.117	0.043	83.429	2.715	0.00805
Pollen diameter	Intercept	−18.380	15.011	84.000	−1.224	0.224220
	Age	0.602	0.139	84.000	4.342	3.92 × 10^−5^
	Age^2^	−0.005	0.001	84.000	−4.059	0.000110
	Mean *T* −4 days	1.840	0.454	84.000	4.054	0.000112
	Mean RH −27 days	0.338	0.137	84.000	2.477	0.015247
Pollen diameter s.d.	Intercept	28.434	11.283	15.492	2.520	0.02314
	s.d. rad −26 days	−0.269	0.080	83.907	−3.337	0.00126
	Mean T −39 days	1.044	0.477	83.443	2.188	0.03146

Abbreviations: Age, relative inflorescence developmental age; s.d. rad, mean standard deviation in light radiation; mean *T*, mean temperature; mean RH, mean relative humidity, followed by the day respective to the moment of flower sampling on which the conditions took place.

Regarding pollen germination rate in cultivars, the overall fit of the model was low. A large part of the variation was again explained by differences between genotypes, whereas only a minor extent was determined by developmental age and environment (conditional *R*^2^ = 0.278, marginal *R*^2^ = 0.092). Only the temperature occurring 40 days before anthesis, i.e. corresponding to initiation of male meiosis, was positively related to the pollen germination rate (Table 3).

The significant relationship between mean pollen diameter and pollen viability observed in *Musa* cultivars (*R* = 0.47, *P* = 3.8 × 10^−17^; Supplementary Data Fig. S4) indicates that pollen size, besides acting as an indicator for gametophytic ploidy, is related to pollen functionality in these backgrounds. To this end, both mean pollen diameter and the associated s.d., as a proxy for the occurrence of larger sized pollen fractions, were modelled (Table 3). Owing to the quadratic term in the model for mean pollen diameter, no conditional *R*^2^ could be obtained; however, the marginal *R*^2^ was relatively high compared with the values of marginal *R*^2^ of other models for the cultivar group, with 0.338 of the variation explained by fixed effects. This includes strong positive effects by the mean temperature level 4 days before anthesis and by mean RH during early microspore development (−27 days). For the s.d. in pollen grain diameter, the observed variation is again largely explained by genotypic differences and only to a small extent by fixed effects (conditional *R*^2^ = 0.702 and marginal *R*^2^ = 0.052). However, some significant environmental effects were observed, with variability in pollen size, hence formation of larger pollen grains, being positively related to the ambient temperature during early meiosis (−39 days), whereas it was negatively linked to radiation in early microspore development (−26 days).

## DISCUSSION

### Developmental time frame of microgametogenesis in *Musa*

Our study provides, for the first time, an approximate time frame of microgametogenesis in *M. acuminata*. Cytological staging of developing male flowers revealed that initiation of meiosis occurs 40 days before anthesis and is completed ∼3 days later (i.e. tetrad stage), namely at 37 days before anthesis. Next, subsequent stages of microgametogenesis were also pinpointed, with PMI occurring between 20 and 15 days before anthesis and PMII taking place 7–5 days before anthesis. This is relatively long in comparison to the 30 days from anther primordium to anthesis in maize (Marchant and Walbot, 2022), but not abnormal, given that in apricot the development of meiocytes into mature pollen takes 30–45 days (Julian *et al.*, 2014). Future screening of microgametogenesis stages in additional *M. acuminata* genotypes is needed to fine-tune the developmental time frame of male reproduction in this *Musa* spp. As an important observation in the present study, DAPI staining of mature *M. acuminata* pollen revealed that these are tricellular at the moment of anthesis, both in greenhouse conditions in Belgium and in the field in Tanzania, and not bicellular as was previously assumed (Brewbaker, 1967). Brewbaker (1967) stated that *Musa* pollen is bicellular, and attributed previous observations of tricellular pollen made by Schnarf (1939) to the use of old pollen. Our cytological data, however, indicate that PMII takes place ≥5 days before anthesis, hence that banana pollen grains are, by definition, trinucleate. This has major implications for the physical properties of the pollen with relevance to the conservation of banana pollen. In general, binucleate pollen undergoes maturation drying, a programmed dehydration process that occurs during pollen development and leads to desiccation tolerance. This allows binucleate pollen to remain viable over a long period of time, though these pollen grains exhibit a lower germination speed compared with trinucleate pollen (Brewbaker, 1967; Lora *et al.*, 2009; Franchi *et al.*, 2011). Trinucleate pollen maintains a higher water content and metabolic activity, because of which it germinates faster but survives for only a short period (Hoekstra and Bruinsma, 1975; Franchi *et al.*, 2011; Williams and Brown, 2018). This aligns with the fact that bananas grow in humid tropical environments, where maturation drying is less necessary, and the observation that banana pollen loses its ability to germinate when dried (Youmbi *et al.*, 2011). Future research should aim to implement cryopreservation protocols designed for short-lived, trinucleate pollen for conservation of banana pollen (Nagar *et al.*, 2023).

From Supplementary Data Table S2 it can be deduced that the frequency of flower cluster opening is genotype dependent. The same was observed by Waniale *et al.* (2021) in a study on flower opening patterns in banana at two different sites in Uganda, with ‘Calcutta 4’ opening a bract every 0.7 days, ‘Mchare’ diploid cultivars and East African Highland Bananas (AAA) opening one bract per day, and Plantain cultivars (AAB) one every 1.5 days. The flower opening frequency might by influenced by environmental factors, because the ‘Calcutta 4’ in greenhouse conditions in this study opened flowers at an exact rate of one per day, whereas in Uganda they opened at a shorter interval of 0.7 days (Waniale *et al.*, 2021). Although the difference in opening rate is minor, it might also be attributable to biological variation, given that Waniale *et al.* (2021) found no effect of ambient temperature or light intensity on bract opening rates in the genotypes in their study.

### Dynamics of pollen viability and germination capacity throughout flowering

The male flowering period in *Musa* is marked by the almost daily production of flower clusters for several months up to 1 year. Our work demonstrates that wild *Musa* accessions showed a rather constant viable pollen percentage of ∼69.1 % and only a slight decrease in pollen germination during flowering. Likewise, Ssebuliba *et al.* (2008) saw a rather high and uniform pollen viability of 70–80 % according to acetocarmine staining, throughout the entire male inflorescence development in wild ‘Calcutta 4’. Despite substantial variation in our viability tests, the data obtained confirm the occurrence of a slight, yet significant, sexual degeneration in wild *Musa* accessions, which is typical for outcrossing plant species that have a long flowering period (Malagon *et al.*, 2019). Notwithstanding this decline in pollen performance, enough potential to achieve successful pollination remains, even at the end of the male flowering phase.

In contrast, the percentage of viable pollen and the germination rate in *Musa* cultivars slightly increased throughout flowering, by 0.068 % and 0.009 % per unit of developmental age, respectively. This was also noted by Krishnakumar *et al.* (1992), who saw an initial increase in pollen germination in cultivars (from 20 % in the first male cluster to 30 % germination in cluster 15); unlike our results, they then observed a sharp decline in germination towards 6 % in cluster 50. Also Ssebuliba *et al.* (2008) saw that triploid East African Highland banana cultivars and the diploid ‘Pisang Lilin’ cultivar exhibit highest pollen viability in the mid clusters of the rachis. This initial increase in fertility is potentially attributed to the transition from female flowers (male infertile), via neutral flowers, towards male fertile flowers (Turner *et al.*, 2024). The findings of Ssebuliba *et al.* (2008) and Krishnakumar *et al.* (1992) do coincide with our finding that most well-formed pollen grains of haploid and diploid sizes (>70 µm diameter) occur immediately after mid-flowering, at relative developmental age 60 of the male inflorescence in cultivars. The fact that we do not observe a downward trend in pollen viability and germination can be a consequence of the relatively short flowering period of most cultivars, where flowering is terminated before degeneration commences. Cultivars used in this study flowered for 4–7 months, whereas wild accessions flowered for 7–14 months (Fig. 1). Also in the study by Ssebuliba *et al.* (2008) cultivars produced ∼100 clusters, whereas wild diploid ‘Calcutta 4’ produced 200 clusters. We hypothesize that the earlier termination of male flowering in cultivars is related to the amount of energy that is reserved towards fruit development. In comparison to wild accessions, in cultivars a considerably larger amount of resources is diverted towards the development of the fruits, and this might negatively impact the sustenance of male flowering and overall pollen quality. Indeed, a study by Dzoyem *et al.* (2023) shows that following removal of female flowers soon after anthesis in cultivars, both the amount of pollen per anther and the pollen viability measured via Alexander’s stain increased significantly.

### Environmental effects on pollen viability and germination

To date, studies on *Musa* pollen viability have taken into account the effects of environmental conditions only on the moment of anthesis (Ortiz *et al.*, 1998; Uma and Arun, 2016). Pollen grains, however, are formed by a lengthy developmental process (Fig. 5) and are impacted by environmental conditions at several stages throughout pollen development (Ito *et al.*, 1970; Saini *et al.*, 1984).

Overall, in wild accessions, both pollen viability and germination are impacted by environmental parameters at several stages throughout microgametogenesis. Especially, the effect of light radiation was reoccurring. Light determines plant growth, morphology and many developmental changes (De Wit *et al.*, 2016) and is thus, in many ways, necessary and beneficial to microgametogenesis (Quedado and Friend, 1978; Xie *et al.*, 2014; Qin *et al.*, 2022). However, too much light can cause accumulation of reactive oxygen species (ROS), which, in severe cases, can cause death and abortion of developing microspores (Hu *et al.*, 2011). In line with this, pollen germination and viability were negatively linked to light radiation during early microspore development, i.e. before spores undergo PMI and are still dependent on the tapetal layer. Precocious induction of programmed cell death in the tapetal layer through ROS accumulation under strong light radiation could prevent crucial transfer of sugar and cell wall components from the tapetal layer to the developing spores, and thereby cause pollen lethality (Hu *et al.*, 2011; Xie *et al.*, 2014). Also at 10 days prior to anthesis, immediately before PMII, radiation showed a negative correlation with pollen germination, and again at 3 days before anthesis to pollen viability, indicating that ROS build-up in the microspore itself is also detrimental to pollen fertility (Muhlemann *et al.*, 2018). Overall, our results show a general negative impact of high light intensity during microspore development on pollen quality in wild *Musa* accessions and thus contrast, in part, with findings by Ortiz *et al.* (1998), who reported that high solar radiation at the moment of anthesis is beneficial to pollen viability in *Musa*. It is likely that plants grown under a high year-round solar radiation, as in the study by Ortiz *et al.* (1998), have an increased antioxidant synthesis to counter the continuously high ROS levels, whereas ROS accumulation in the plants in our study might have had more severe effects from sudden increases in light intensity, affecting sensitive microspore stages (Szymańska *et al.*, 2017; Liu *et al.*, 2019). An increase in temperature immmediately before PMII was positively related to germination, and during PMII it was positively related to viability. A positive effect of temperature was expected, because *M. acuminata* is native to the humid (sub)tropics (De Langhe *et al.*, 2009) and coincides with the positive relationship seen by Ortiz *et al.* (1998) between temperature at the moment of anthesis and pollen viability in wild bananas and cultivars in the field in Nigeria. For the same reason, a negative effect of mean RH during meiosis and between PMI and PMII on germination was unexpected, although also in the study by Ortiz *et al.* (1998) a high minimum humidity (during anthesis) decreased mature pollen viability. High relative air humidity (RH > 85 %) is known to deregulate the balance between plant CO_2_ uptake and water loss (Torre *et al.*, 2003). Mean RH was on average 80 % during our study, but could occasionally reach ≤100 %, thereby possibly stressing the plants and impacting developing microspores. Given that the effects of environmental conditions on different stages of microgametogenesis on corresponding pollen performance observed in this study are inferred from correlations, future experimental work will have to determine whether the observed relationships can be confirmed via controlled exposure to certain environmental stresses. Especially, ROS accumulation during pollen development under high light intensities should be monitored in banana and related to the viability of the mature pollen.

In *M. acuminata* cultivars, we see a different response to environmental variables during pollen grain development compared with wild accessions, with less impact of radiation and more of temperature. Meiosis is clearly the most sensitive stage in terms of environmental effects on eventual pollen characteristics, whereas later stages during gametogenesis are not affected as much. Elevated temperatures during meiosis are correlated with increases in viable pollen percentage, germination percentage and s.d. in pollen size, indicative of increased formation of larger pollen grains. The association of larger pollen grains with higher temperatures aligns with previous studies that report enhanced production of unreduced (2*n*) pollen in banana cultivars upon exposure to high solar radiation and increased temperatures (Ortiz *et al.*, 1998). This 2*n* pollen has an increased size owing to its increased DNA content. Although the occurrence of 2*n* pollen has not been demonstrated in the cultivars in our study, the observed correlation between temperature stress during meiosis and the eventual formation of enlarged pollen suggests instances of meiotic restitution and associated formation of 2*n* pollen. Given that meiotic restitution forms a natural route to restore the otherwise aneuploid karyotypic status of spores produced by triploids towards viable triploid gametes (Murthy and Tiwari, 1987), its induction in specific environmental conditions could explain the associated improvement in pollen viability. However, further in-depth analyses are needed to unravel the mechanistic link between enlarged pollen size and environmental (temperature) stress and whether and to what extent this contributes to improved pollen performance in *Musa* cultivars.

### Conclusion

Male meiosis takes place in *M. acuminata* ssp. 40–37 days prior to anthesis. Pollen mitosis I and II take place between 20 and 15 days and between 7 and 5 days before anthesis, respectively. *Musa acuminata* pollen therefore appears to be tricellular at anthesis. Inflorescence developmental age and environmental conditions during male gametogenesis affect pollen performance simultaneously and should both be monitored to improve pollen quality. In wild accessions, pollen viability and germination gradually decrease throughout inflorescence development, whereas in cultivars the viability and germination improved slightly towards the end of flowering. Most well-developed pollen grains of haploid size or larger in cultivars were found slightly after the middle of the male inflorescence development. Pollen viability and germination in wild accessions were impacted negatively by increased light intensity during meiosis, before and during PMI, during PMII and several days before anthesis. Therefore, pollen quality in wild accessions might benefit when plants are sheltered from high light intensities. Pollen viability, germination and size were increased by elevated temperatures during meiosis in *M. acuminata* cultivars, implying that there might be potential for improvement of male fertility in cultivars by application of a heat treatment during meiosis.

## Supplementary Material

mcaf268_Supplementary_Data

## Data Availability

The data underlying this article are available in KU Leuven RDR, at https://rdr.kuleuven.be/dataset.xhtml?persistentId=doi:10.48804/OVQG25.

## SUPPLEMENTARY DATA

Supplementary data are available at *Annals of Botany* online and consist of the following. Figure S1: fluorescein diacetate (FDA) intensity plot of freshly harvested pollen versus pollen treated for 1 h at 70 °C. The red line at intensity of 0.07 separates dead pollen (below) from viable pollen (above). The freshly harvested pollen consists of all data assembled from the *Musa accuminata* ssp. *malaccensis* inflorescence that flowered from November 2020 to January 2022 (11 120 measured pollen grains). The heat-treated pollen was collected from the same plant, but from a different inflorescence on 12 November 2022 (3024 measured pollen grains). Figure S2: violin plots for pollen diameter over subsequent sampling moments for *Musa acuminata* ssp. *malaccensis* (A) and triploid AAA cultivar ‘Williams Bell’ (B). The black arrowhead indicates the date on which the accompanying bright-field pictures on the right were taken. White arrowheads indicate aborted pollen, and the white arrow indicates larger pollen (than the standard haploid size). Figure S3: images show representative nuclear configurations of DAPI-stained mature pollen nuclei, harvested and fixed in the field in Arusha, Tanzania. Tricellular pollen grains of *Musa acuminata* ssp. *malaccensis* (A–D), *Musa acuminata* ssp. burmannica ‘Calcutta 4’ (E–H), AAcv ‘Pitu’ (I–L), AAcv ‘Malaysian blood’ (M–P) and AAcv ‘Uwati’ (Q–T). Figure S4: correlation analysis between the mean values of the three pollen quality parameters (i.e. viability, germination rate, and pollen diameter) acquired over all dates for both cultivars and wild accessions. (A, B) Correlation between mean germination percentage and mean viable pollen percentage for wild accessions (A) and cultivars (B). (D, E) Correlation between mean pollen diameter and mean viable pollen percentage for wild accessions (C) and cultivars (D). (E, F) Correlation between mean pollen diameter and mean germination percentage for wild accessions (E) and cultivars (F). Corresponding *P*- and *R*-values are shown in the plot. Table S1: feed solution for 100 L stock solution (diluted to 10 000 L). The feed solution supplied nutrients at EC 1.7 and a pH of 5.5. Stocks A and B were mixed immediately before administration to prevent precipitation of components. Table S2: bract opening rates for the genotypes under study, calculated as the total days of the male flowering phase divided by the total number of nodes on the rachis at the end of flowering. n.a. denotes missing data where only one inflorescence was monitored for a genotype and no mean and s.d. in bract open rate could be obtained.
