# Supplementary figures and images for: Combined effects of inflorescence developmental age and environmental conditions during microgametogenesis on pollen viability, germination and size in Musa acuminata ssp
Source: Ann Bot. 2025 Oct 23;137(2):557–70. doi: 10.1093/aob/mcaf268 (PMC12823240; doi:10.1093/aob/mcaf268)

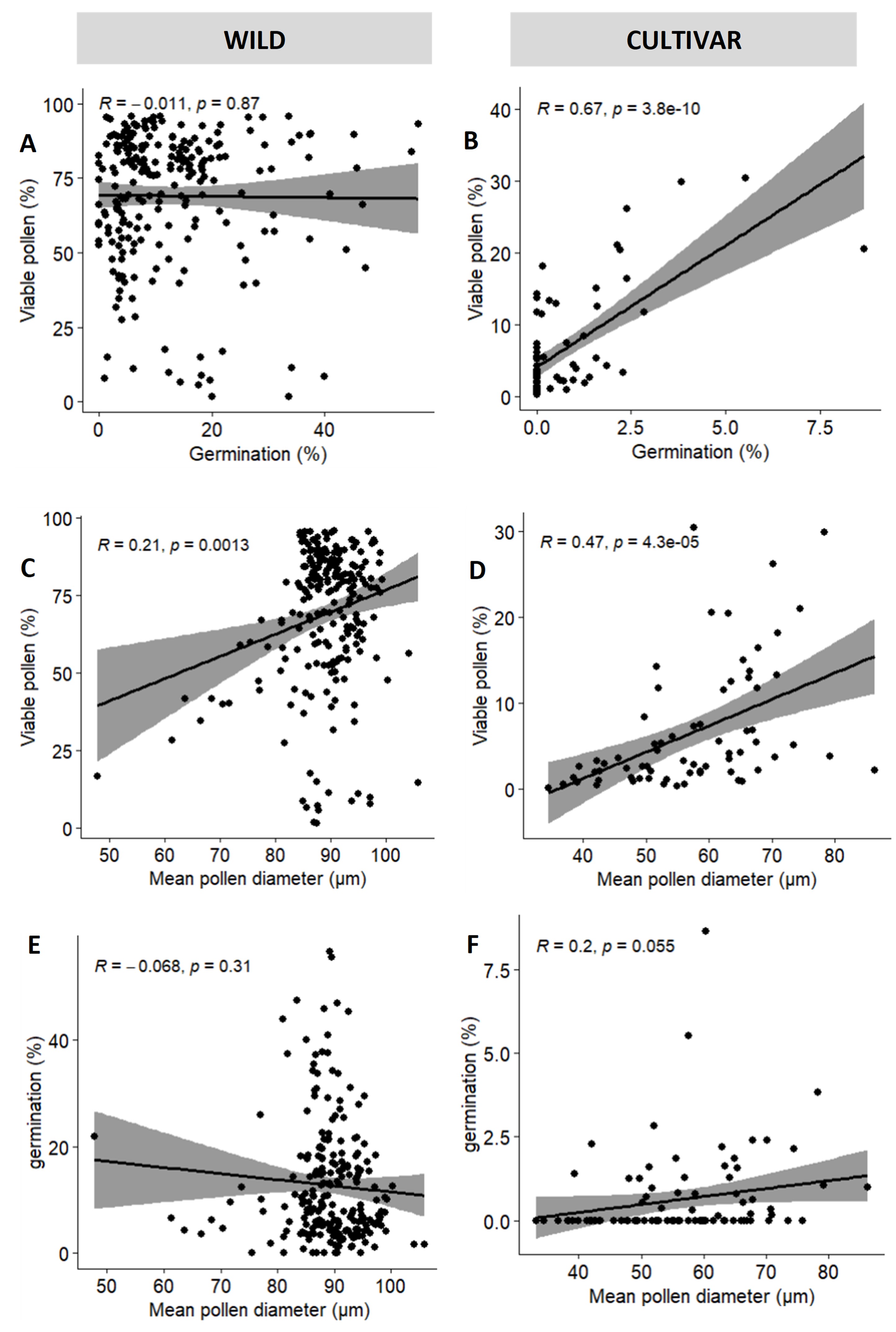

Supplement: mcaf268_Supplementary_Data [file mcaf268_supplementary_data.zip › Figure_S4.jpg]

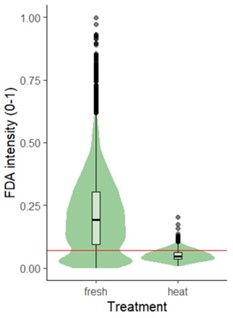

Supplement: mcaf268_Supplementary_Data [file mcaf268_supplementary_data.zip › Figure_S1.jpg]

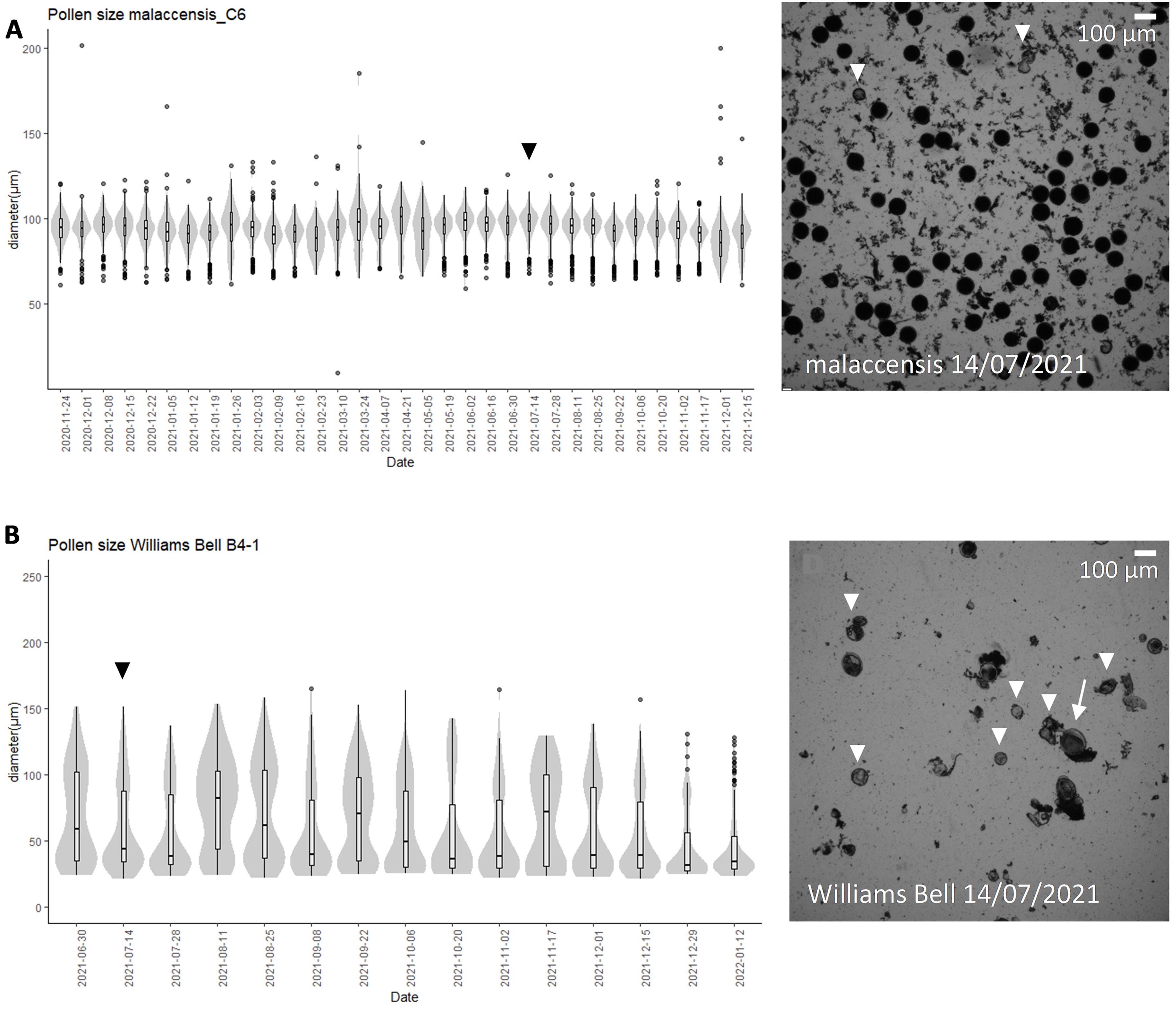

Supplement: mcaf268_Supplementary_Data [file mcaf268_supplementary_data.zip › Figure_S2.jpg]

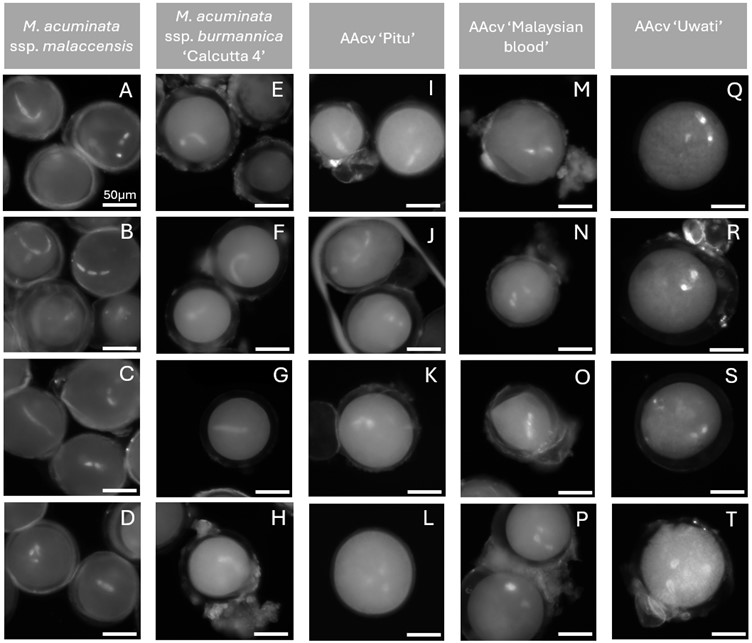

Supplement: mcaf268_Supplementary_Data [file mcaf268_supplementary_data.zip › Figure_S3.jpg]
